# A nursing process for self-management support in home healthcare: A qualitative study

**DOI:** 10.1016/j.ijnsa.2026.100551

**Published:** 2026-05-05

**Authors:** Åsa Audulv, Nathalie Wiklund, Linnea Lönneborg, Anna Larsson Gerdin

**Affiliations:** a Department of Nursing, Umeå University, Umeå, Sweden; b Department of Health Sciences, Mid Sweden University, Sundsvall, Sweden

**Keywords:** Home healthcare services, Nurses, Patient education as topic, Qualitative research, Self-care, Self-management

## Abstract

**Background:**

Nurses play a key role in providing self-management support to people living with long-term health conditions. Home healthcare patients often face difficulties self-managing because of disabilities and multi-morbidities. In addition, providing healthcare in patients’ homes comes with specific challenges since the healthcare providers have less control over the care environment. Therefore, home healthcare is an ideal setting for investigating how nurses provide self-management support in complicated situations.

**Aim:**

This study focuses on the process and activities nurses undertake when providing self-management support to home healthcare patients, developing a model to elaborate and clarify nurses’ self-management support work.

**Methods:**

In this interpretive description study, 24 registered nurses, working with home healthcare services, took part in qualitative semi-structured interviews. Interviews were performed in two rounds (in 2019 and 2024). During the second round the nurses commented on and helped further develop the model illustrating the findings.

**Results:**

This study presents a Nursing Model of Self-management Support Provision. Part one, shows how nurses’ working conditions and individual belief systems impact their ability and willingness to provide self-management support. Part two, describes the nurses’ clinical judgement and strategies used to provide self-management support. In summary, nurses described how their employing organization created barriers to self-management support, due to lack of guidelines, limited time and training. Differing belief systems also influence practices, nurses being deliberate in providing self-management support used a wider range of supports and could adapt them to the patients’ situation. Nurses assessed patients’ risks and potential for self-management before deciding whether to provide self-management support. Most nurses described using a relatively limited number of supports. Common support included teaching and giving advice, supporting sustainable behaviour change, and support to manage other resources. Finally, the nurses evaluated patients’ self-management performance to decide whether patients should continue to self-manage, needed more support, or return to assisted healthcare.

**Conclusions:**

The developed model can be used to clarify nurses’ self-management support activities, for example in teaching or in planning self-management support initiatives. Consistent with previous research, the nurses identified information as the most used self-management support strategy. To provide sufficient self-management support nurses’ need 1) a working environment that facilitates self-management support, 2) adopt a wide range of self-management support strategies, and 3) move beyond a traditional medical understanding of self-management.

**What is already known**

• Nurses often struggle to implement self-management support.
• Providing self-management support in home healthcare is especially complicated due to patients’ health status, often including additional disability and comorbidities.

**What this paper adds**

• Nurses’ self-management support activities and clinical judgement have been described in a model that includes patient assessments, support provision, and evaluation.
• The developed model clarifies the process nurses use to provide self-management support.
• To provide sufficient self-management support, nurses need to have organizational opportunities, be deliberate in the self-management support they provide, adopt a wide range of self-management support strategies, and move beyond a traditional medical understanding of self-management.

Alt-text: Unlabelled box dummy alt text

## 1 Background

### 1.1 Self-management and self-management support

Individuals’ ability to self-manage long-term health conditions and their consequences is vital to mitigate symptoms and maintain overall health (Allegrante et al., 2019). Self-management is recognized as strategies and activities that people (and their families) undertake to live well with long-term health conditions (Audulv et al., 2016; Barlow et al., 2002). The most well-known aspect of self-management is medical management, which includes following treatment regimens and mitigating symptoms (Bonetti et al., 2022; Yang et al., 2022). However, self-management also includes managing roles and emotions by adapting daily life to a long-term condition, to maintain leisure activities, relationships, and wellbeing despite persistent symptoms or specific health needs (Audulv et al., 2019). Because people experience different symptoms and life circumstances, individuals experiment and tailor strategies to their own needs and abilities (Audulv et al., 2021).

Self-management support includes systems, policies, services and programs undertaken to strengthen people’s capacity to self-manage and provided by health, social or community agencies. It can be integrated into healthcare consultations, or provided in comprehensive programs, in person, or digitally (Mills et al., 2017), with registered nurses often playing a central role (Been-Dahmen et al., 2015; Coster et al., 2020). While self-management support varies, many approaches include disease and treatment information, support for lifestyle changes, development of self-management action plans, symptom self-monitoring, and strategies to enhance problem solving skills (Dineen-Griffin et al., 2019). However, while nurses recognize the importance of self-management support, they can struggle to implement it in their work (Dwarswaard and van de Bovenkamp, 2015; van Het Bolscher-Niehuis et al., 2020). For example, self-management support is not consistently recognized as a core nursing responsibility by nurses or other professionals (Tharani et al., 2025), nurses conceptualization of self-management support varies (Been-Dahmen et al., 2015; Hämel et al., 2022), and nurses often experience limited organizational support for providing self-management support (Tharani et al., 2021, Tharani et al., 2025). Nurses tend to focus their self-management support on the medically oriented aspects of self-management, while strategies for managing role and emotional aspects of illness are often overlooked (Been-Dahmen et al., 2015). Provision of self-management support can present ethical dilemmas for nurses, when patients’ right to autonomy conflicts with decisions that challenge healthcare recommendations (van Het Bolscher-Niehuis et al., 2020; Wilkinson et al., 2016). There is a lack of research studies describing nurses’ clinical reasoning or judgements regarding when and how nurses provide self-management support to patients. Clinical judgement is cognitive reflections and reasoning processes that proceed a clinical conclusion (Connor, et al., 2023), including for example assessing patient’s needs, reasoning regarding care alternatives, and making decisions about actions (Tanner, 2006).

### 1.2 Self-management support in home healthcare settings

Demographic shifts and evolving social policies have led to a growing number of older adults receiving healthcare at home. Home care services are a vital component of the healthcare system in many countries, though its organization varies (Genet et al., 2012). In Sweden municipalities are responsible for home care services, including both home healthcare regulated by the Health and Medical Act (SFS 2017:30), and home help services (such as help with meals, cleaning, grocery shopping, and personal hygiene performed by home care aids) as outlined in the Social Services Act (SFS 2001:453). In Sweden, home healthcare is mainly directed towards older adults who are housebound, about 4% of the population receiving these services (The Swedish National Board of Health and Welfare, 2023). Home healthcare services are often delivered by interprofessional teams in which registered nurses are the largest professional group, working alongside physiotherapists, and occupational therapists which focus on home rehabilitation (Johansson et al., 2023; Saint-Pierre et al., 2018). Home healthcare nurses’ work includes providing medical treatments (e.g., injections or organizing pills), managing wound care, parenteral nutrition, monitoring chronic conditions, care planning, and documentation (Fjørtoft et al., 2021). They collaborate with the patients’ general practitioners (employed by the regional healthcare centres), which are responsible for prescribed medical treatment.

The home setting provides unique opportunities to provide self-management support, care is embedded in patients’ daily routines, and nurses can individualize their support to align with patients’ abilities and preferences (Dowding et al., 2020). However, providing healthcare at home poses challenges, for example limited control over the care environment, working alone, and ethical considerations regarding patients’ autonomy and integrity (Larsson Gerdin and Hellzén, 2021). The provision of self-management support can also be constrained by a task-oriented care model where professional control dominates, and patients are perceived as passive recipients (Larsson Gerdin et al., 2024a). Moreover, older adults who receive home healthcare frequently face challenges to self-management, including physical or cognitive limitations, complex treatment regimens, and/or limited self-efficacy (Tian et al., 2024). Home healthcare patients are likely to become dependent on receiving regular home healthcare, while, in fact, research indicates that this group seeks greater autonomy and many older adults want to perform treatments independently (Larsson Gerdin et al., 2024b). Unfortunately, there is limited research exploring how nurses perceive and engage in self-management support with their patients when caring for them in their homes.

### 1.3 Study rational

In home healthcare, there are aspects that facilitate self-management support, like long-term caring relationships, care provided in the patients’ home environment, and nurses possibilities to autonomous decision-making. At the same time home healthcare patients have traditionally been viewed as being care dependent, typically struggling with disability and/or cognitive impairment. Taken together, this makes home healthcare an ideal setting to investigate how nurses use clinical judgement and provide self-management support. Current research has mainly focused on barriers and ethical dilemmas, overlooking what nurses do and how they make clinical judgements regarding self-management support. Understanding nurses’ clinical judgement processes is vital to create education and interventions. Therefore, this study focused on the process and activities nurses undertake when providing self-management support to home healthcare patients and developed a model to clarify nurses’ self-management support work.

## 2 Method

### 2.1 Design

This study uses an Interpretive Description approach. Interpretive Description focus on investigating clinically relevant problems, for example conceptual models usable in practice (Thorne, 2016). Reporting follows the Consolidated Criteria for Reporting Qualitative Research (COREQ) (Tong et al., 2007) (see supplementary file 1).

### 2.2 Settings and recruitment

Home healthcare nurses were recruited in eight municipalities representing rural areas, smaller towns (∼50.000 inhabitants), and middle-sized cities (∼100.000 inhabitants) in central and northern Sweden. Contacts were established with the municipalities’ home healthcare managers, who in turn invited nurses to take part in the study. The managers supplied the research team with contact information to interested nurses, which received information from the research team, both verbally and in writing, before deciding about participation.

To be eligible, participants had to be a registered nurse (e.g., have a bachelor’s in nursing), and a minimum of six months’ work experience in home healthcare. No exclusion criteria were used. Purposive sampling considered gender, urban versus rural district, and length of work experience.

In total, 24 registered nurses participated, of whom 21 were women and three men. About half (n = 13) had specialist education at master’s level in district nursing. Experience working in home healthcare ranged from six months to 20 years (md 6 yrs); experience as a nurse ranged from 3 to 35 years (md 17). Most (n = 17), worked solely as a visiting nurse (both planned and acute). Two had combined positions working part-time doing home healthcare visits and part time in nursing homes. Three nurses worked solely evenings and weekends responding to urgent or unplanned care needs. The last two participants worked mostly administratively to organize home healthcare services and admitting patients into the home healthcare system, granting and planning services.

### 2.3 Data collection

Data collection was performed in two rounds. First, semi-structured interviews were performed with 19 nurses (April to September 2019). Later, five additional nurses were interviewed to further inform the analysis (October to November 2024). The second round of interviews was conducted both to investigate if self-management support had changed during these five years, and to test the interpretation of the preliminary results. In Interpretive Description, this process is called ‘the thoughtful practitioner test’ and assists development of results relevant to clinical practice (Thorne, 2016).

Most interviews were performed face-to-face at the home healthcare nurses’ offices, a few were conducted over the telephone, depending upon the preference of the participant. The first round of interviews was conducted either by ÅA, NW, or LL. The second round of interviews were performed by ÅA and ALG together. ÅA and ALG have substantial experience of qualitative interviewing. The interviews performed by NW and LL contributed to their training and they received supervision (e.g., interviews were performed together with ÅA and transcripts were read to provide feedback). NW and LL, who work in home healthcare, did not interview anyone with whom they had a working relationship.

The interviews focused on the self-management support provided by the nurses, and their understanding of patients’ needs and difficulties regarding self-management. Interview questions were open ended, and probes were used to encourage participants to further develop and add depth to their answers (see supplementary file 2: Interview Guide). The interview guide was pilot tested before use. The second round of interviews included two parts, the first part followed the interview guide from the earlier interviews. In the second part, preliminary findings were presented and the participants were asked to elaborate on the model. Interview length varied between 20 and 120 min, with most about 40 min in length. Interviews in the second round were in general longer. The interviews were audio recorded and transcribed verbatim.

### 2.4 Analysis

The analysis was performed in four phases: comprehending, synthesizing, theorizing, and recontextualizing (Thorne, 2016). In the comprehending phase, interview transcripts were read, and data were coded with a focus on nurses’ perception of self-management, and self-management support. In the synthesizing phase, the focus shifted to exploring patterns in the data. During this phase, the different roles nurses took regarding self-management support were identified, as well as various self-management support strategies. In the theorizing phase, we posed analytical questions to the data, for example we investigated how the different roles were related to certain self-management support barriers. Thus, we were looking for relationships between the different categories. In the last phase, the recontextualization phase, we developed “a Nursing Model of Self-management Support Provision”, a model connecting all categories.

The comprehending phase was conducted by NW and LL, supervised by ÅA. The later phases were conducted by ÅA with input from ALG.

### 2.5 Ethical considerations

All nurses gave informed, voluntary consent before participating. The study adhered to international legislation regarding research ethics including the Helsinki Declaration (World Medical Association, 2018), the European (All European Academies, 2023) and Swedish (Swedish Research Council, 2024) Code of Conduct for Research Integrity. According to Swedish research ethics law (SFS 2003:460) all research should be performed according to good research practice, but a formal research ethics application to the Swedish Ethical Review Authority is not required for studies that do not collect sensitive personal data (defined in the law as data related to specific personal aspects of health, religion, or ethnicity) (Swedish Research Council, 2024). In this study the collected data focused on nurses’ work practices, non-sensitive personal data. Therefore, according to Swedish law ethics approval was not required.

## 3 Findings - A nursing model of self-management support provision

**Fig. 1:**
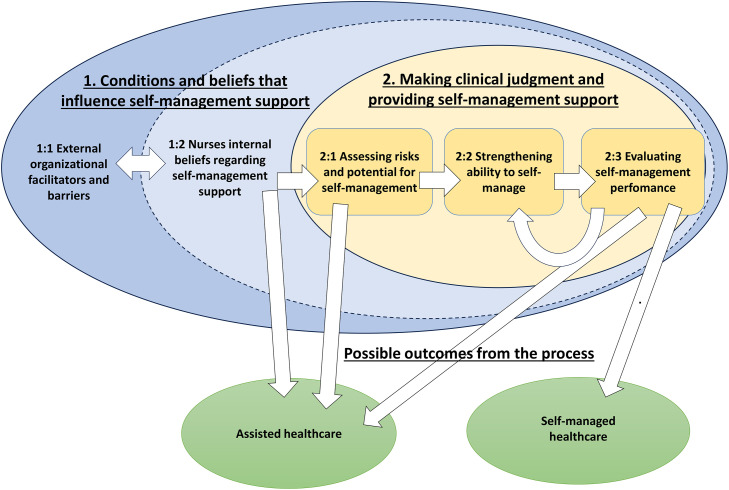
A Nursing Model of Self-management Support Provision. The figure displays how the different parts of the model are related to each other.

A Nursing Model of Self-management Support Provision describes how nurses working in home healthcare support patient self-management. The model contains two parts: Part one describes the conditions and beliefs that impact nurses’ ability and willingness to provide self-management support, and Part two describes the nurses’ clinical judgement and strategies they use to provide self-management support. The model also shows two possible outcomes, either the nurses could continue to provide “assisted healthcare”, or they could support the patient to do “self-managed healthcare”. Assisted healthcare represents the type of care nurses mostly provide in home healthcare, meaning that nurses perform activities for patients, such as wound-dressing, injections, or preparing oral medication. Self-managed healthcare was when the patients could take over the responsibility for healthcare activities and perform them independently or with support from family. The healthcare activities described were mostly related to medical management, role and emotional management were described to a small extent (such as supporting social activities). The parts of the model are intertwined since nurses’ working conditions and their own beliefs impact on how they approach the self-management support they provide (see Fig. 1).

### 3.1 Part one: conditions and beliefs that influence self-management support

The nurses described conditions that either facilitated or posed barriers to provision of self-management support. They described organizational barriers, such as limited time and lack of training, but also how their own belief systems (e.g., how they understood self-management support and their own role in providing it) impacted their way of approaching self-management support. The nurses’ belief system also seemed to influence how they interpreted organizational barriers. For example, nurses who emphasized interest in and dedication to self-management support tended to describe fewer organizational barriers and more facilitators.

#### 3.1.1 External organizational facilitators and barriers

The nurses described that their own organizations and the wider healthcare system impacted their possibility of providing self-management support, mostly posing barriers. This was attributed to focus on instrumental tasks, with no specific time allocated for patient education or self-management support. Nurses described how teaching a procedure demanded a longer visit than performing the procedure themselves. Still some nurses thought that teaching patients to help themselves would save time in the long run. None knew of guidelines for providing self-management support at their unit, or on a regional level. Patient expectations often made provision of self-management support more difficult. This was particularly problematic when discharge planning included information suggesting patients would receive treatment at home, rather than support patients to learn treatment routines.

I would like to have more structure in my work [with self-management support], I think it is about giving equal opportunities for all that receive our support. […] I believe I miss a template for what we should provide. (Sara)

None of the nurses described having specific training in self-management support, but some described receiving training in motivational interviewing which they found helpful. Specialist nurses described having more experience encouraging behaviour change and these experiences were considered helpful when adapting self-management support to different patients.

The home healthcare units were organized in different ways. In some units’ nurses described working in teams. In other units the nurses worked mostly alone, with sole responsibility for a specific number of patients. The nurses working alone described limited discussions about self-management support with colleagues, the individual nurse did not know what strategies for self-management support colleges used. In contrast, nurses working in a team described discussing self-management support of individual patients within the team. Problem-solving and information sharing were also easier when nurses had a close working relationships with allied health professionals, physicians, and home care aids. The nurses described that home care aids could be especially important since they met the patients on a day-to-day basis and could approach the nurse when problems occurred. Working relationships were, for example, facilitated by shared office space and lunchrooms.

#### 3.1.2 Nurses’ internal beliefs regarding self-management support

Beliefs related to self-management support influenced nurses’ practices. Beliefs varied regarding what self-management included, why, when and for whom it was (or wasn’t) important, as well as the barriers/facilitators most prevalent for home healthcare patients. These beliefs could be sorted into three belief systems which represented perceived roles when providing self-management support, each with a different level of engagement (see Table 1 for more detail).

**Table 1:** This matrix shows how the three self-management support roles represent various belief systems.

	**Beliefs and perceived barriers related to the three different perceived self-management support roles**		
	**Role #1: “Providing deliberate self-management support”:**	**Role #2: “Providing non-reflective self-management support”:**	**Role #3: “Not my job”:**
Beliefs about self-management support	Self-management support is seen as a central part of care. Nurses have a key role in providing self-management support. Use of planned and deliberate self-management support activities: - providing information, - teaching practical skills, - encouraging and gradually letting patients take over tasks, - individually tailoring follow up, - lining patients with other care providers and resources.	Self-management support is seen as important but as an intuitive and unplanned part of care. Self-management support was provided simultaneously with other work tasks. Use of intuitive and unplanned self-management support activities: - giving advice and information - teaching practical skills	Self-management support is not seen as part of home healthcare working description. Self-management support should preferably be provided by primary healthcare centers. Use of self-management support when prompted by direct questions: - giving information - referring patients to specialized healthcare providers.
Perceived barriers to self-management support	**Working situation:** Self-management support takes time and commitment; nurses need to be able to be engaged in order to provide good support. **The healthcare organization:** Limited communication and care planning across providers can lead to missed information and opportunities. It is unclear how self-management support should be documented in patient records. **Patient characteristics:** Patients can be unsure about performing a task, have little knowledge and motivation. Cognitive impairment or mental illness are potential barriers to self-management. **Social aspects:** Patients might want a visit from home healthcare nurses, as it provides company and feelings of being cared for. Therefore, some patients might not want to take on tasks themselves.	**Working situation:** Lack of time, stress and unclear expectations were barriers to providing self-management support. Self-management support had a low priority in relation to other work tasks. **The healthcare organization:** healthcare providers (especially when patients are hospitalized) might incapacitate patients by taking over management. **Patient characteristics:** Patients in home healthcare are often fragile, with cognitive impairment. Limited disease knowledge can also be a barrier.	**Working situation:** The working schedule was tight and there was no time to take on additional tasks like self-management support. **The healthcare organization:** Self-management support should foremost be provided by the healthcare provider responsible for medical care. **Patient characteristics:** Patients receiving home healthcare services have limited capacity for self-management, often because of cognitive impairment. **Patients’ health risks:** It is important that medical procedures are performed the right way. Patients’ health could be at risk if the patients themselves performed procedures inconsequently or wrongly.

In **the first role, “Providing deliberate self-management support”** (described by nine specialist nurses) self-management support was described as a central part of the care provided. These nurses described self-management as a means for patients to help themselves, thus becoming more independent. Self-management was also seen as important for healthcare providers work situation; if patients could self-manage their care, or part thereof, healthcare resources could be spared. These nurses planned for self-management support and actively taught and motivated patients to develop self-management. They emphasized the importance of multidisciplinary teams and described how they functioned as a link between the patients and other care providers and resources. Further, both home care aids and family were valued as important resources in supporting or assisting self-management.

We would like to help the patients to manage as much as possible themselves, because then we will get fewer consultations […] Depending on how well you can support self-management, and give advice, and assistance to relatives, patients, and home care aids… so we try to help them all, so the patient can become more independent, manage more in their home and become less reliant upon us. (Erik, role 1)

**Role two, “Providing non-reflective self-management support”** was described by eleven nurses including both nurses and specialist nurses. They described their self-management support as intuitive and unplanned, in contrast to role one. Role two nurses described self-management as important but stressed many barriers. Self-management support was often provided at the same time as the nurses performed other tasks, for example, advice was integrated in a conversation with the patient. These nurses adapted information and support according to the situation but did not seem to reflect on or plan the support they provided, some nurses gradually became more aware of the self-management support they provided during the research interview.

It [self-management support] isn’t anything I plan beforehand, instead you get to read the situation, and see how much they [the patients] can pull off and how much they would like [to take responsibility for] and such. (Maria, role 2)

**The third role, “Not my job”** was described by four nurses who said that self-management support was not really part of their job description. In contrast to role one and two, the nurses describing role three did not provide self-management support by their own initiative, only when directly asked. From the “Not my job” beliefs, self-management was described as important for certain groups of patients (such as people with diabetes) but that support should be provided by the healthcare provider responsible for their medical care, such as the patient’s diabetes nurse or general practitioner. One nurse explained that she found home healthcare patients unable to self-manage and therefore she did not provide self-management support:

Well… the patients we work with, they often need help with everything. It’s clear… it would be different at a healthcare center where you deal with relatively healthy and able people, but I do not have any experience of that. (Alice, role 3)

### 3.2 Part two: making clinical judgement and providing self-management support

The nurses described three phases when making clinical judgement and providing self-management support; i) Assessing risk and potential for self-management, ii) Strengthening ability to self-manage, and iii) Evaluating self-management performance. The process had two possible outcomes, either a continuation of assisted healthcare or a shift to self-managed healthcare.

How nurses engaged in clinical judgement and provided self-management support depended on what role they took. Nurses in “Role 1: Providing deliberate self-management support” were most deliberate in their clinical judgement and provided more complex and adapted self-management support strategies. Those in “Role 2: Providing non-reflective self-management support” described all parts of the process, but clinical judgement was done by intuition, and they described less variation in the self-management support strategies they provided. Finally, those in “Role 3: Not my job” mainly identified barriers and risks, thus mostly continued with assisted healthcare.

#### 3.2.1 Assessing risk and potential for self-management

Assessment of patient risk and potential for self-management support were key to clinical judgement for nurses in home healthcare. The assessment included three aspects: assessing potential risks or health hazards associated with the activities, assessing the patient’s potential ability, and lastly assessing the type of self-management support needed. The nurses described assessing patient characteristics such as hand function, cognitive function, and interest in learning about their disease. One nurse described assessments:

Certainly, you do have a discharge report from the hospital, but most times they [the patients] are in bad shape [at discharge]. They might have had an operation, and still have opioids in their system, and be rather perplexed. One thing could be written in my papers… […] and it could be a big difference when he is at home and has become better. (Eva, role 2)

Assessing risks and potential for self-management occurred mostly through observation, or during ‘small-talk’ with the patient, family members and/or home care aids. Possible cognitive impairment could complicate the assessment. When the nurses suspected that the patient had little motivation, or cognitive impairment they were reluctant to support self-management. While nurses expressed a desire to honor patients’ choices to be independent or take responsibility, doing so when they felt there were risks to patients’ health or outcomes was difficult, and a middle ground was often sought. One nurse described how patients with suspected cognitive impairment could be allowed to maintain responsibility for less potent medications.

I have had someone, for example, that has had medication in a pill box… that [medication] might be less time dependent, and it might be little difference if you miss a dose. Then you may let this person manage it by themselves, because you should not tread on somebody’s toes. Overall, the most important thing is that you get to perform what you can yourself, and feel that you are in charge, and [that you] are involved in managing your care. (Madeleine, role 2)

Depending upon the assessment, nurses either decided to support the patient’s self-management by strengthening self-management ability, or they continued with assisted healthcare.

#### 3.2.2 Strengthening ability to self-manage

Some nurses knew of and used a variation of self-management supports, while others only described a few. Role 1 nurses described several types of support and a more nuanced understanding of how and when each support should be provided. The nurses had not received any formal training in providing self-management support, therefore their individual clinical experiences directed what support they provided. Three categories of support were described: Teaching and giving advice, Supporting sustainable behaviour change, and Supporting a network of resources (See Table 2).

**Table 2:** Three categories of self-management support described by nurses in home healthcare settings.

**Strengthening ability**
**Teaching and giving advice** − Answering questions − Giving advice and information − Providing written information − Adapting advice − Teaching procedures
**Supporting sustainable behaviour change** − Giving reassurance and motivating change − Encouraging responsibility − Creating routines and structure − Creating goals
**Supporting a network of resources** − Supporting family care-givers self-management assistance − Collaborating with home care aids − Collaborating with other health services − Giving advice regarding clients’ healthcare navigation − Connecting clients to community services

While the nurses’ main focus was directed at patients’ medical management (e.g., taking medication, disease knowledge, and performing treatment procedures), nurses also described supporting healthy behaviours (e.g., exercise, losing weight or smoking cessation), and a few described that they supported role and emotional self-management, for example related to mental health, and social isolation.

**Teaching and giving advice:** Teaching was a common form of self-management support. It took the form of didactic, one-way communication, or open discussion adapted to the patients’ level of knowledge and circumstances. Nurses often described teaching procedures (e.g., how to give subcutaneous injections, dress a wound, or prepare infusions). They described starting by showing the procedure and then gradually letting the patients try until the patients independently mastered the procedure.

So [you give] information, you show, and they get to try. You may need to visit a few times, and they get to show how it is done, so they feel comfortable. And then after that you follow up and have regular contact to see that it gets done. (Minna, role 2)

In a few cases nurses described providing written information (e.g., medical guidelines or information leaflets). One nurse also described using teach-back technique, where she first showed a procedure then asked the patient to demonstrate or show her.

The nurses described teaching activities in the first and the second round of interviews. In the second round some nurses described looking up information on their smart phone while in the patients’ home or showing short instruction videos to their patients and relatives. In the quote a nurse describes how she used an online video resource to teach a patient and his family members about an inhalation device. The situation was complex since the family was immigrants with limited Swedish.

Then I thought… should I get an interpreter, or what should I do? But I went onto the medication-instructions website and found a really good video. So, I showed them the video, and then they got to assemble these parts [for the inhalator device] until they could put the parts together, and they knew where to insert the medication [into the inhalator]. (Ingrid, role 1, interview round 2)

**Supporting sustainable behaviour change**: The nurses, especially Role 1 nurses, tried to motivate, encourage, and provide skills for behaviour change and to maintain self-management over time. Some described using communication strategies inspired by motivational interview technics. Nurses encouraged patients’ self-efficacy and tried to motivate patients to take active responsibility for their health. Nurses also helped patients to create routines for self-management or discussed self-management goals. For example, they advised patients to exercise at the same time each day, and they introduced aids (e.g., pillboxes, lists or medication distribution robots) to support routines for taking medications.

One important part is to reinforce self-confidence. To be able to do it yourself. It might actually be the difference between feeling very exposed, like being totally dependent and then maybe feel like I can contribute myself… I can influence my life and situation, despite having multimorbidity… I can do it. (Evelyn, role 1)

**Supporting a network of resources:** Nurses described how they worked to link and manage the resource system around the patients. They described teaching and assisting family caregivers (e.g., spouses or adult children), collaborating with home care aids and other healthcare services, connecting patients to social services and supporting the patients’ health navigation. Health navigation support included giving advice on where to seek further support or assisting patients to locate and understand information on healthcare web-pages. One nurse told about helping patients apply for special transportation service.

I print a form, and bring to you, and then we fill it out together, and then you put it in an envelope and mail it. Because then I help them, but they get to apply themselves. It is not me applying for them. (Filippa, role 1)

#### 3.2.3 Evaluating self-management performance

The nurses evaluated patient’s health situation and self-management performance during follow up. When unsure of a patient’s ability, they checked in more regularly - in person, over the phone or by asking home care aids to check on specific aspects. As part of the evaluation nurses reassessed self-management potential for changes or improvements. This generally resulted in one of three decisions, 1) the patient could continue to self-manage the procedure, 2) the patient needed more self-management support, 3) the risk of poor outcomes remained very high despite the patient’s desire or attempts to self-manage the treatment/procedure and the nurse reintroduced assisted healthcare. One nurse described a situation where she had supported a patient to self-manage their leg ulcer:

Then it is important that I have a follow up too, and that I let them know. Like ‘then I will call you or visit in about two weeks and see how it has changed.’ (Evelyn, role 1)

## 4 Discussion

Nurses have a key role in providing self-management support, still they often struggle with this work (Tharani et al., 2021; Beaudin et al., 2025; Wuyts et al., 2021). In this study, we developed a Nursing Model of Self-management Support Provision to better understand how and under what conditions nurses provide self-management support. The model describes how organizational barriers/facilitators and nurses’ own beliefs systems create conditions for how nurses’ approach self-management support. The second part of the model describes the process of providing and evaluating self-management support. The model can be used to clarify and plan nurses’ work, for example to make organizational changes and train nurses. In this study, home healthcare is the setting in which the nurses worked. However, the general parts of the model are likely transferable to other healthcare settings, with the specifics being more context bound. For example, all nurses and healthcare providers are likely to have a belief system regarding self-management and related concepts, but their particular beliefs probably differ between regions and healthcare organizations.

The Results show several barriers to self-management support, such as lack of guidelines, limited training and nurses’ own beliefs that self-management is too difficult for their patients. A large systematic review of barriers and facilitators to self-management support described similar barriers, including 1) nurse-related factors, like competence and interest in self-management support, 2) patient related factors, like motivation and cognitive impairment, 3) relationship related factors, such as building rapport and reciprocity, and 4) factors related to the organization and healthcare system (Tharani et al., 2021). Within our model, the barriers and facilitators to self-management support exist at different levels, for example, some are related to organizational aspects and others to the nurses’ skills in providing self-management support. Pinpointing where the barriers lie is important for organizations that wish to embark on initiatives to reduce barriers. For example, addressing lack of guidelines requires a different approach than skill development (e.g., Wuyts et al., 2021).

In clinical judgement, nurses draw on different kinds of knowledge, of which the nurses’ previous experience, beliefs and knowledge are the most important (Tanner, 2006). In the current study, the nurses’ perceived role was both the starting point for how they approached provision of self-management support, but also how they conceptualized and emphasized barriers/facilitators. This aligns with previous research on clinical judgement. Tanner (2006) describes how nurses’ various beliefs, for example of health or moral right, impact their clinical judgement and ultimately their decisions and actions. The nurses providing deliberate self-management support had a pattern of being more nuanced regarding barriers and facilitators to self-management support. Many nurses in this study were not deliberate in the self-management support they provided and therefore decisions made were not explicitly informed by evidence. The dual process model describes two different cognitive processes in clinical reasoning; processes can either be intuitive or analytical. Intuitive processes are heavily reliant on pattern recognition. Nurses would recognize the clinical situation from previous experience and knowledge and make a conclusion without being aware of the cognitive process. In contrast, analytical processes include logical reasoning regarding alternatives and possible outcomes. An analytical process takes more time and might be most important when there are high-stake outcomes, uncertain and complex situations. However, intuitive and analytical processes are also often used in combination (Pelaccia, et al., 2011; Quaresmal, 2019). In light of the dual process model, the nurses describing role 1 (deliberate self-management support) emphasized analytical processes as part of their clinical judgement, that likely helped them see alternatives and adapt their self-management to the patient’s situation. In contrast, nurses describing role 2 (non-reflected self-management support) integrating self-management support into other care activities, that might support self-management support being performed. However, because role 2 nurses did not describe reflecting about self-management support or making deliberate decisions they might have a reduced ability of tackling more complex situations or developing their self-management support skills. A precondition for analytical processes is knowing that alternatives exist and reflecting on the best way to proceed. As Molina-Mula and Gallo-Estrada (2020) suggest, nursing decisions are shaped not only by personal reflection but also by institutional norms. Without sufficient time for reflection or consideration of alternative approaches, nurses may default to routine practices instead of exercising their full professional judgment or working to their full scope of practice.

It was evident that the nurses had various belief systems regarding self-management and thus took on different roles in providing support. Possible explanations appear to be related to work culture, different training/education, and/or individual experience and motivation. van Hooft et al., 2024 suggested that hospital nurses are hampered in providing self-management support due to working routines which emphasize instrumental tasks and their own expectations, focus on patients’ medical needs and are usually focused on the short-term. Similarly, a recent study (Tharani et al., 2025) shows ambiguities between nurses’ expectations and work description, for example was patient education part of the work description but not integrated in the daily work. Nurses in home healthcare also described expectations and unclear work descriptions as barriers.

The home healthcare nurses directed most of their support activities towards medical management and treatment, overlooking self-management directed towards role and emotional management. On one hand, this could be related to the organization and structure of home healthcare services, which for the most part is focused on providing medical treatments in patients’ homes. On the other hand, previous research representing other healthcare contexts has also described how nurses tend to emphasize the medical parts of self-management (Otter et al., 2021, van Hooft et al., 2024). Home healthcare patients often experience physical impairment, as well as mental health problems and social isolation, all of which can negatively impact their overall well-being in old age (Courtin and Knapp, 2017) suggesting they would likely benefit from more comprehensive self-management support. For example, self-management support directed towards managing health risks, engaging in healthy behaviours, reducing loneliness, and improving mental health seems indicated. Home healthcare nurses could play a more significant role in preventing illness among this group by widening their scoop of practice, as has previously been shown in research concerning integrated primary care (Beaudin et al., 2025).

Previous research describes nurses experiencing dilemmas when weighing the risk of poor health outcomes against respecting patient autonomy (Otter et al., 2021; van Het Bolscher-Niehuis et al., 2020; Wuyts et al., 2021). Similarly, the nurses in this study did not include the patients’ opinions in their assessment of self-management potential, despite shared decision-making being the norm in Swedish healthcare legislation. It is possible that nurses in home healthcare gatekeep more than nurses in other healthcare contexts, because cognitive impairment is common among patients in home healthcare settings and is known to complicate decision-making. Still, research shows that patients receiving home healthcare services describe being persuaded to hand over responsibility for treatments to nurses, despite wanting to be more involved (Larsson Gerdin et al., 2024). Future research should investigate how assessing potential for self-management can include both the perspective of nurses, patients and relatives. In the current study, some nurses did describe overcoming the dilemma between risks of poor health outcomes and patient autonomy by gradually letting the patient take over a treatment task, and/or individually tailoring follow up. In such cases, the nurse had the opportunity to intervene if a serious health risk occurred. In these cases, collaboration between the nurse and the home care aids appeared important, since the home care aids met the patients every day and could inform the nurse if problems arose. An individually tailored follow-up might be crucial in healthcare context with frail patients whose health and self-management capacity can rapidly change.

The nurses descriptions about self-management support varied. Since the units lacked guidelines and none of the participants had specific training in self-management support, each nurse had developed their own ways of supporting self-management. Some innovative nurses used activities such as the teach-back method, adapted motivational interview technics, and/or on-line resources, but they were the exceptions. Most nurses described using few support activities, and activities widely known in the self-management research literature seemed to be unknown to the nurses in this study. For example, activities like creating action plans, monitoring symptoms, and goal-setting were hardly mentioned. The most used self-management support activity in this study was giving information, which aligns with previous research (Dineen-Griffin et al., 2019; Molina-Mula and Gallo-Estrada, 2020; Otter et al., 2023). However, it should be noted that information alone is often insufficient to support behaviour change (Vernooij et al., 2016). This implies a gap between self-management research evidence and the knowledge used in clinical practice. In the current study, there was a pattern between the nurses’ type of training and engagement in self-management support. In general, specialist nurses were more deliberate in providing self-management support and described more varied self-management support activities, suggesting that training plays an important role in how nurses perceive and provide self-management support. An extensive literature review showed that both nurses and nursing students felt unprepared to provide self-management support (Wuyts et al., 2021), which in turn suggests that education initiatives are needed.

### 4.1 Implication for practice

Organizations need to prioritize self-management support if it is to be promoted as part of nurses’ work. The model presented here can be used by organizations to identify areas in need of change. Conditions such as training, clear guidelines, and time are needed for nurses to work with self-management support (McGowan, 2013; Vallis, 2015). However, managers might also need to work with nurses’ beliefs systems, so they want to and feel equipped to provide self-management support (Vallis, 2015).

Because the model clarifies the process of providing self-management support it holds potential to inform education and training. For example, assessment of self-management ability and ways to promote self-management, such as coaching and counselling are areas for inclusion in curricula. Further, organizations should develop guidelines to ensure that self-management performance is properly evaluated.

Home healthcare nurses are in an ideal position to support self-management and work preventatively, by raising awareness of risks of falls, healthy eating, and giving advice regarding mental health. It is possible that if nurses had the possibility to work more holistically, they could prevent some of the acute and costly health crises that can befall older adults. This would demand that nurses in home healthcare are allowed to broaden their scope of practice, which currently is task oriented and focused on patients’ medical needs and treatments. This is an important area for further research.

### 4.2 Strengths and limitations

In total, 24 nurses were interviewed. However, since the nurses were invited by their managers, we cannot determine how many potential participants declined participation. It should be noted that inviting employed healthcare providers to participate in research by contacting their managers is standard procedure in Sweden, especially if the data collection is performed during working hours. However, the managers did not get any information about which nurses that eventually took part and names in the article are pseudonyms. To include different kinds of experiences we recruited participants from eight different municipalities, with varied working experiences. Most interviews were long and rich in content and aspects that were not initially planned as part of the interview guide were often brought up by participants. One of the aspects that emerged from the data and became an important part of the findings was the concept of clinical judgement.

We chose not to send transcripts to participants for review and approval to ensure consistency in the data analysis process, as participants’ feedback on transcripts could potentially introduce variability or bias that might complicate the interpretation of the findings. However, since the interviews were transcribed verbatim soon after the interview sessions, we consider this approach sufficient to ensure the accuracy and reliability of the data. In addition, round two provided opportunity for additional participant feedback on model development.

This study started in 2019, and most interviews were conducted at that time. Since both the COVID pandemic and increased digitalization have impacted home healthcare settings, we complemented the initial data collection with a second round of interviews in 2024. However, the interviews from the two rounds of data collection showed a remarkable similarity, which indicate no substantial difference in nurses work with self-management support in home healthcare. In cases where we found differences, they are described in the result section, most notably the use of smartphones to aid self-management support. During the second round of interviews, a preliminary version of the model was presented to nurses, and they agreed that it captured their way of working and clarified their working process. Input from these interviews resulted in minor changes to the model.

Healthcare is organized in various ways across the world, and municipality organized home healthcare services are largely unique to the Nordic countries. However, homebased healthcare is conducted in many countries, especially in those with developed healthcare systems and an aging population (Al-Hamad, 2025). When comparing our results to research from other healthcare context there are many similarities in how nurses experience working with self-management support (Otter et al., 2021; (Tharani et al., 2025).

## 5 Conclusions

As in other areas of healthcare, self-management support in home healthcare is not yet clearly defined in terms of guidelines or role expectations, leaving nurses to manage with limited resources. This study presents a model for how nurses within the context of home healthcare use clinical judgement and provide self-management support. The model can be used to clarify the process and thus make responsibilities and decisions more visual for nurses and/or other healthcare providers supporting self-management. Further, the model showed that barriers and facilitators to providing self-management support are related to both the organization and the individual, this is important to identify barriers and develop ways of overcoming them. To provide sufficient self-management support nurses need to 1) be deliberate in the self-management support they provide, 2) use varied self-management support activities, and 3) move beyond a traditional medical understanding of self-management. This model was developed from the nurses’ perspectives. Future research should consider home healthcare patients’ needs but also focus on the systems in which the nurses work. Home healthcare patients often have multiple healthcare contacts and future research should detangle what self-management support could be provided in which part of the healthcare system. To develop self-management support interventions for home healthcare patients, both patients, nurses and the healthcare system need to be taken into account.

## Funding

During 2019, this study was funded by a collaboration grant from Sundsvall Municipality and Mid Sweden University (ÅA). After 2019 the research did not receive any specific grant from funding agencies in the public, commercial, or not-for-profit sectors.

## Declaration of generative AI and AI-assisted technologies in the writing process

During the preparation of this work the authors used M365 Copilot in order to evaluate some English terms and synonyms, in addition, English terms have also been discussed with native English speakers. After using this tool, the authors reviewed and edited the content as needed and take full responsibility for the content of the publication.

## Availability of data and material

The dataset generated and analyzed in the current study are not publicly available because participant consent included restrictions on use of the data due to patients’ privacy concerns. Limited availability is possible. Researchers wishing information should contact Associate Professor Åsa Audulv (asa.audulv@umu.se).

## CRediT authorship contribution statement

**Åsa Audulv:** Writing – original draft, Visualization, Supervision, Resources, Project administration, Methodology, Investigation, Funding acquisition, Formal analysis, Data curation, Conceptualization. **Nathalie Wiklund:** Writing – review & editing, Investigation, Formal analysis, Data curation. **Linnea Lönneborg:** Writing – review & editing, Investigation, Formal analysis, Data curation. **Anna Larsson Gerdin:** Writing – review & editing, Visualization, Validation, Investigation, Formal analysis.

## Declaration of competing interest

The authors have no conflicting interests.

## Appendix Supplementary materials

Image, application 1

Image, application 2

## Footnotes

Supplementary material associated with this article can be found, in the online version, at doi:10.1016/j.ijnsa.2026.100551.
