# Supplementary material for: A nursing process for self-management support in home healthcare: A qualitative study
Source: Int J Nurs Stud Adv. 2026 May 5;10:100551. doi: 10.1016/j.ijnsa.2026.100551 (PMC13157178; doi:10.1016/j.ijnsa.2026.100551)
Supplement: Supplementary file 1 [file mmc1.docx]

## **Supplementary file 1: The Consolidated Criteria for Reporting Qualitative Research: Check-list**

| **Domain 1: Research team and reflexivity Personal Characteristics** | | **Part in paper where this aspect is addressed** |
| --- | --- | --- |
| 1. Interviewer/facilitator (Which author/s conducted the interview or focus group?) | The first round of interviews were performed by either ÅA, NW or LL. ÅA has an extensive experience of conducting qualitative interviews, NW and LL were in training and received supervision on how to interview. The second round of interviews were performed by ÅA and ALG together. | Data collection, author contribution |
| 2. Credentials (What were the researcher’s credentials? E.g. PhD, MD) | ÅA is a Reg Nurse, PhD and Associate Professor in Nursing. NW and LL are Reg Nurses and have MSc in primary care nursing. ALG is a Reg Nurse, Post doc-fellow, and Senior Lecturer in Nursing. | Authors bio |
| 3. Occupation (What was their occupation at the time of the study?) | ÅA and ALG are employed by universities, both having combined teaching and research positions. NW and LL were, during the data collection doing their master degrees, but have since then worked as nurses in primary care and home healthcare. | Authors bio |
| 4. Gender (Was the researcher male or female?) | All authors are women. | Authors bio |
| 5. Experience and training (What experience or training did the researcher have? Relationship with participants?) | ÅA has extensive experience of conducting and teaching qualitative methods and have conducted several studies with an Interpretive Description approach. ALG have experience in qualitative research. NW and LL took part in this study as part of learning research and qualitative methods. LL and NW, who work in home healthcare, had worked on the same home care teams as some of the participants, however they did not interview any of their former colleges. | Authors bio  Data collection |
| 6. Relationship established (Was a relationship established prior to study commencement?) | The potential participants received information about the study and had a telephone conversation with the researchers previous to the interviews. | Data collection  Research Ethics |
| 7. Participant knowledge of the interviewer (What did the participants know about the researcher? e.g. personal goals, reasons for doing the research) | The participants received information that the research group were nurses and interested in self-management support in home healthcare, that ÅA had previously conducted self-management research and that NW and LL were students doing a master. | - |
| 8. Interviewer characteristics (What characteristics were reported about the interviewer/facilitator? e.g. Bias, assumptions, reasons and interests in the research topic?) | ÅAs research area is self-management, but most often from a patient perspective. She generally uses a broad definition of self-management including both medical, role and emotional management of long-term conditions. NW and LL have and are working in home healthcare organizations. ALGs research is in understanding dilemmas and premises both for nurses and patients in providing/receiving home healthcare. | Author bio |
| **Domain 2: study design Theoretical framework** | |  |
| 9. Methodological orientation and Theory (What methodological orientation was stated to underpin the study? e.g. grounded theory, discourse analysis, ethnography, phenomenology, content analysis) | Interpretive description [1]. Dimensions of self-management were defined in alignment with the Taxonomy of Everyday Self-management Strategies (TEDSS) [2]. | Background  Design |
| 10. Sampling (How were participants selected? e.g. purposive, convenience, consecutive, snowball) | A purposive sample was used. We strived for variation in municipality, length of working experience, rural/urban location, and gender. | Data collection |
| 11. Method of approach (How were participants approached? e.g. face-to-face, telephone, mail, email) | Managers at several different home health-care units were approached, some managers agreed to ask if their staff were willing to participate in the study. Possible participants then received an information letter by e-mail and a phone call with further information. | Setting and recruitment |
| 12. Sample size (How many participants were in the study?) | First round of interviews: 19 nurses  Second round of interviews: 5 nurses | Data collection |
| 13. Non-participation (How many people refused to participate or dropped out? Reasons? Setting?) | Because of the recruitment strategy we could not determine how many potential participants that got informed about the study but chose not to participate. | Limitation section |
| 14. Setting of data collection (Where was the data collected? e.g. home, clinic, workplace) | Participants were interviewed in person at their place of work or over the phone. | Data collection |
| 15. Presence of non-participants (Was anyone else present besides the participants and researchers?) | No non-participants were present during interviews. | - |
| 16. Description of sample (What are the important characteristics of the sample? e.g. demographic data, date Data collection) | 1^st^ data collection took part between April and September in 2019.  2^nd^ data collection was conducted during October and November in 2024.  Sample characteristics is described in the paper. | Data collection |
| 17. Interview guide (Were questions, prompts, guides provided by the authors? Was it pilot tested?) | The interview guide was piloted with minor revisions. Interview guide is attached as a supplementary file. | Data collection |
| 18. Repeat interviews (Were repeat interviews carried out? If yes, how many?) | No repeat interviews were used. | Limitation section |
| 19. Audio/visual recording (Did the research use audio or visual recording to collect the data?) | Audio recordings were used. | Data collection |
| 20. Field notes (Were field notes made during and/or after the interview or focus group?) | Field notes were not used. | - |
| 21. Duration (What was the duration of the interviews or focus group?) | Interviews varied from 20 to 120 minutes, with most about 40 minutes in length. | Data collection |
| 22. Data saturation (Was data saturation discussed?) | We used ideas about information power in qualitative studies [3] to decide on appropriate number of participants in order to collect a varied and rich data set focusing upon our research question. Data saturation is not a concept used in Interpretive Description. | - |
| 23. Transcripts returned (Were transcripts returned to participants for comment and/or correction?) | Transcripts were not returned to the participants. | - |
| **Domain 3: analysis and findings Data analysis** | |  |
| 24. Number of data coders (How many data coders coded the data?) | NW and LL did the preliminary data coding, the analysis was later continued by ÅA. | Analysis |
| 25. Description of the coding tree (Did authors provide a description of the coding tree?) | In accordance with interpretive description [1] we did not use a detailed coding three, but instead sorted the material in larger preliminary categories. These categories were resorted a number of times before the final conceptual model was constructed. | - |
| 26. Derivation of themes (Were themes identified in advance or derived from the data?) | The analysis was primarily inductive. | Analysis |
| 27. Software (What software, if applicable, was used to manage the data?) | The analysis was performed in Word. | - |
| 28. Participant checking (Did participants provide feedback on the findings? Reporting?) | Preliminary findings were presented and discussed at two conferences with nurses working in home health-care and later the second data collection provided additional data on the interpretation and emerging conceptual framework. | Data collection, Analysis, Limitations |
| 29. Quotations presented (Were participant quotations presented to illustrate the themes / findings? Was each quotation identified? e.g. participant number) | Quotations are used to illustrate and exemplify the findings. Participants have pseudonyms to indicate how many participants are quoted. | Findings |
| 30. Data and findings consistent (Was there consistency between the data presented and the findings?) | Quotations are chosen to show how the data aligns with the findings. | Findings |
| 31. Clarity of major themes (Were major themes clearly presented in the findings?) | Major themes are described in the findings section. | Findings |
| 32. Clarity of minor themes (Is there a description of diverse cases or discussion of minor themes?) | Investigating contrasting cases was an important part of the analysis and the findings include various examples. Some minor themes are also further detailed in tables. | Findings |

1. Thorne, S., *Interpretive Description – Qualitative research for applied practice.* 2nd ed. 2016, New York: Taylor & Francis.

2. Audulv, Å., et al., *The Taxonomy of Everyday Self-management Strategies (TEDSS): A framework derived from the literature and refined using empirical data.* Patient Education and Counseling, 2019. **102**(2): p. 367-375.

3. Malterud, K., V.D. Siersma, and A.D. Guassora, *Sample Size in Qualitative Interview Studies: Guided by Information Power.* Qual Health Res, 2015: p. 1049732315617444.
