# Supplementary material for: A nursing process for self-management support in home healthcare: A qualitative study
Source: Int J Nurs Stud Adv. 2026 May 5;10:100551. doi: 10.1016/j.ijnsa.2026.100551 (PMC13157178; doi:10.1016/j.ijnsa.2026.100551)
Supplement: Supplementary file 2 [file mmc2.docx]

# Supplementary file 2: Interview guide

(Translated from Swedish. The interviews included probing adapted to the interview situation. Probes focused on developing thoughts further, give different examples and/or describe the underlying meaning of examples.)

**Introduction – work situation and self-management generally**

- Could you describe your work?
  - What kind of unit do you work at?
  - Short about your every-day work tasks…
- If you would describe self-management for a nursing student, how would you do that?
- If you would describe self-management for a patient, how would you do that?
- Is self-management important for the patients you meet? In what way?
- What kind of self-management do you think your patients need?
- If you would think about a patient that you’ve recently met that has problems self-managing in every-day life, could you describe that situation? Why do you think it is difficult to manage for this patient?
- If you would think of a patient, you’ve met that performed self-management well, could you describe that situation? What do you think, support and facilitates for that patient?

**Working with self-management support**

- Do you believe that self-management support is more important for specific groups of patients? Which? Why would self-management support be more important for them?
- Would you say that you work with self-management support in your daily work? Could you tell me about that?
- How do you plan your work with self-management support?
  - Do you use any particular communication strategy? For example, motivational interviewing or cognitive support. Tell me about it…
  - Do you use any kind of instrument? (for example digital devices, information folders) Tell me about it…
  - Do you use any kind of screening tool or questioner to assess patients’ self-management or self-management ability?
  - Do you participate in or lead any kind of patient education program? Tell me about it…
- How do you go about to find out what kind of self-management your patients do, or if they have problems with their self-management?
  - Do you support your patients to find resources, problem solve or find information? Tell me about it…
- Are there barriers in your work that make your work regarding self-management support harder? Tell me about it…
- Do you talk about how to support self-management among your colleges? Tell me about it… How does it differ between professions?

**Ending the interview**

- Have you attended any training or education regarding self-management support?
  - Would such training be needed? Why? Why not? What would you like to learn about?
- Is there something more about self-management or self-management support, that you would like to tell me?
